# A New Approach to the Etiology of Syncope: Infection as a Cause

**DOI:** 10.3390/v17030427

**Published:** 2025-03-15

**Authors:** Branislav Milovanovic, Nikola Markovic, Masa Petrovic, Vasko Zugic, Milijana Ostojic, Milica Dragicevic-Antonic, Milovan Bojic

**Affiliations:** 1 Institute for Cardiovascular Diseases “Dedinje”, 11000 Belgrade, Serbia; 2 Faculty of Medicine, University of Belgrade, 11000 Belgrade, Serbia

**Keywords:** syncope, head-up tilt test, autonomic dysfunction, neurocardiology

## Abstract

Background/Objectives: Syncope is a common clinical occurrence, with neurally mediated and orthostatic types accounting for about 75% of cases. The exact pathophysiological mechanisms remain unclear, with recent evidence suggesting autonomic nervous system damage and a potential infectious etiology. This study aimed to examine the role of infection in the development of syncope and orthostatic hypotension (OH). Methods: The cross-sectional study included 806 patients from the Neurocardiological Laboratory of the Institute for Cardiovascular Diseases “Dedinje”. Patients were divided into three groups: unexplained recurrent syncope (n = 506), syncope with OH during the head-up tilt test (HUTT) (n = 235), and OH without a history of syncope (n = 62). All participants underwent the HUTT, and 495 underwent serological testing for various microorganisms. Data were analyzed using chi-squared tests and binary and multinomial logistic regression. Results: The HUTT was positive in 90.6% of patients with syncope and OH, compared with 61.6% with syncope alone (*p* < 0.001). Serological testing revealed that 57.85% of syncope patients, 62.9% of syncope with OH patients, and 78% of OH patients had positive IgM antibodies to at least one microorganism. Multivariate analysis indicated that IgM antibodies to Coxsackievirus and Epstein–Barr virus were significant predictors of OH. Conclusions: This study demonstrated a potential association between infections and syncope/OH. Further investigation into the role of infectious agents in autonomic dysfunction is warranted to clarify the underlying mechanisms of syncope and OH.

## 1. Introduction

Syncope, a common occurrence in clinical practice, is defined by the European Society of Cardiology (ESC) as transient loss of consciousness (TLOC) caused by cerebral hypoperfusion, characterized by sudden onset, short duration, and spontaneous complete recovery [1]. While the etiology of cardiogenic syncope is well understood, the precise mechanisms underlying neurally mediated and orthostatic syncope remain under investigation. According to studies, neurally mediated and orthostatic syncope account for approximately 75% of all syncope cases [2]. A central feature in both is damage to the autonomic nervous system (ANS), leading to disturbances in blood-pressure and heart-rate regulation. ANS dysfunction is well-documented in primary neurological diseases, diabetes mellitus, and kidney damage; however, the cause of this dysfunction in a larger number of patients still remains unclear [3,4,5].

Adler et al. proposed that dysautonomia is a key aspect in patients with Lyme disease [6], consistent with other studies linking Lyme disease and autonomic dysfunction [7,8]. Additionally, the literature reports the occurrence of postural orthostatic tachycardia syndrome (POTS) after infection, with prevalence rates as high as 41%. POTS, syncope, and other forms of dysautonomia have also been observed following infections such as COVID-19 and Epstein–Barr virus [9,10,11,12,13]. Carod-Artal described ANS damage in various infections as a potential mechanism contributing to increased mortality [14]. Furthermore, it has also been suggested that the autonomic ganglia may serve as a site for viral latency [15,16]. These findings raise the question of whether infectious agents contribute to ANS damage, dysautonomia, and, ultimately, syncope.

Orthostatic hypotension (OH) frequently occurs in individuals with recurrent syncope. Studies have previously documented OH in patients with acute infections and orthostatic intolerance in patients with post-COVID-19 syndrome [6,17,18].

The aim of this study was to investigate infection as a potential cause of syncope and OH.

## 2. Materials and Methods

The cross-sectional retrospective review study included 806 patients aged 18 years and older who were examined at the Neurocardiological Laboratory of the Cardiology Clinic at the Institute for Cardiovascular Diseases “Dedinje” from November 2021 to December 2023. Patients were divided into three groups. The inclusion criteria for the first group were patients with unexplained, recurrent syncope. The second group consisted of patients with unexplained, recurrent syncope who exhibited orthostatic hypotension (OH) during the head-up tilt test (HUTT), and the third group included patients without a prior history of syncope who exhibited OH during the HUTT (Table 1).

In all three groups, exclusion criteria were as follows: syncope of cardiogenic etiology (e.g., conduction disorders, arrhythmias, congestive or ischemic heart disease, valvular heart disease, or cardiomyopathy), confirmed epilepsy as a cause of transient loss of consciousness (TLOC) (as diagnosed by a neurologist), OH due to volume depletion or medication use, and patients with primary autonomic neuropathies (e.g., multiple system atrophy, pure autonomic failure, or Parkinson’s disease). Patients with conduction disorders on 24 h Holter ECG monitoring that met criteria for pacemaker implantation, supraventricular tachycardia (including atrial fibrillation/flutter) as the cause of syncope and meeting ablation criteria, or ventricular tachycardia (sustained/nonsustained) were also excluded.

All patients in the study underwent the HUTT using the Westminster protocol at the Neurocardiological Laboratory. Serological testing was performed for 495 patients by a reference institution in Serbia.

This study was supported by grant 451-03-68/2020-14/200156 from the Ministry of Education, Science, and Technological Development of the Republic of Serbia and grant COVANSA from the Science Fund of the Republic of Serbia. This study was performed in line with the principles of the Declaration of Helsinki.

### 2.1. Head-Up Tilt Test (HUTT)

The Westminster protocol was used in this study [19]. Patients spent 10 min in a supine position before the passive phase. The tilt angle was 70 degrees, and the test duration was 45 min. The test was considered positive if syncope or severe presyncope occurred with a drop in blood pressure or the onset of bradycardia. Blood pressure was monitored continuously (Task Force), as was the 12-lead surface ECG.

During the HUTT, other significant hemodynamic changes were recorded, including:

- Extreme variation in blood pressure during the supine or passive phase: Sustained variation where the absolute difference between maximum and minimum systolic pressure values exceeded 20 mmHg.
- Small variation in blood pressure: Sustained variation where the absolute difference between maximum and minimum systolic pressure was between 10 and 20 mmHg.
- Hypertensive reaction: Sustained blood-pressure values exceeding 130/90 mmHg during the passive phase.
- Extreme hypertensive reaction: Sustained blood-pressure values exceeding 170/120 mmHg during the passive phase.
- Postural orthostatic tachycardia syndrome (POTS): A rapid increase in heart rate of more than 30 beats per minute or a heart rate exceeding 120 beats per minute within 10 min of standing, without OH, but with symptoms of orthostatic intolerance [20].
- Orthostatic hypotension (OH): A progressive and sustained drop in systolic blood pressure of more than 20 mmHg from baseline, or a drop in diastolic blood pressure of more than 10 mmHg, or systolic blood pressure falling below 90 mmHg [1].
- Bradycardia during the supine phase: Heart rate < 60 beats per minute (bpm).
- Tachycardia during the supine phase: Heart rate > 100 bpm.
- Hypotension during the supine phase: Blood pressure ≤ 90/60 mmHg.
- Hypertension during the supine phase: Blood pressure > 130/90 mmHg.

### 2.2. Serology Testing

Serological testing for microorganisms was performed in one of the two reference institutions in the Serbian Military Medicine Academy—The Institute of Virology, Vaccines and Sera “Torlak” or the Institute of Public Health “Dr. Milan Jovanovic Batut”. The reference institution selected the appropriate serological test for each microorganism. Samples were collected approximately 3–5 days after patients were tested at the Neurocardiological Laboratory. Test results were categorized as positive or negative.

The serological tests included:

1. Viruses: Herpes simplex virus 1 (HSV1), Herpes simplex virus 2 (HSV2), Varicella-zoster virus (VZV), Cytomegalovirus (CMV), Epstein–Barr virus (EBV), Human herpesvirus 6 (HHV6), Adenovirus, Parvovirus B19, Coxsackievirus, and SARS-CoV-2.
2. Bacteria: *Mycoplasma pneumoniae*, *Chlamydia pneumoniae*, *Coxiella burnetii*, *Bartonella henselae*, *Brucella*, *Toxoplasma gondii*, *Borrelia* spp. (ELISA and Western blot), *Helicobacter pylori*, and *Yersinia enterocolitica*.
3. Fungi: *Candida albicans* and *Aspergillus fumigatus*.

### 2.3. Statistical Analysis

Results are presented as counts (percentages). Bivariate analysis of categorical data between groups was performed using the chi-squared test. Univariate and multivariate binary logistic regression was conducted to evaluate the relationship between dichotomous dependent and independent variables. Multinomial logistic regression was also performed to assess the relationship between nominal dependent variables with three or more categories and independent variables. Prediction models were formed following multicollinearity rules (tolerance coefficient > 0.1 and VIF < 10) and Pearson’s R coefficient (r < 0.7) between independent variables. Outliers were removed using Mahalanobis and Cook’s distance. All *p* values < 0.05 were considered significant. Data were analyzed using SPSS 29.0 (IBM Corp. Released 2022. IBM SPSS Statistics for Windows, Version 29.0. Armonk, NY: IBM Corp.).

## 3. Results

### 3.1. Head-Up Tilt Test

Low blood pressure in the supine position was more prevalent in group 2 (n = 26, 11.1%) and group 3 (n = 8, 12.3%) compared with group 1 (n = 24, 4.7%) (*p* < 0.01; *p* < 0.05). The HUTT was significantly more likely to be positive in group 2 (n = 213, 90.6%) than in group 1 (n = 303, 61.6%) (*p* < 0.001) (Table 2). Extreme variations in blood pressure were more common in group 1 (n = 238, 47%) and group 3 (n = 35, 53.8%) than in group 2 (n = 79, 33.6%) (*p* < 0.01 for both comparisons). Hypertensive reactions were observed more frequently in group 1 (n = 91, 18%) than in group 2 (n = 3, 1.3%; *p* < 0.001) and group 3 (n = 3, 4.6%; *p* < 0.01).

### 3.2. Serological Testing Results

**Figure 1:**
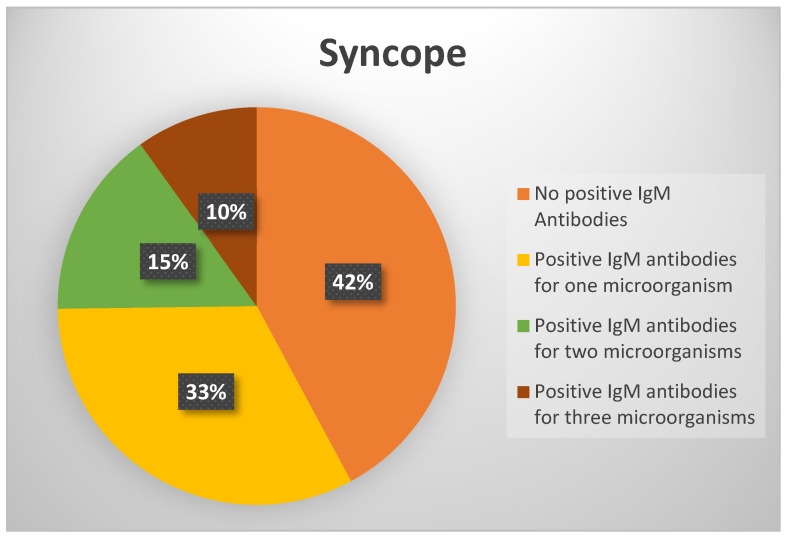
Positive IgM antibodies for group 1.

Complete serological testing results were available for 495 subjects: 242 from group 1, 194 from group 2, and 59 from group 3. In group 1, 102 subjects (42.15%) did not have positive IgM antibodies for any microorganism, while 140 (57.85%) tested positive for at least one microorganism (Figure 1).

The highest percentage of positive results was for IgM antibodies to *Borrelia* spp. (n = 35, 14.5%) and HSV1 (n = 30, 12.4%) (Table 3 and Table 4).

**Figure 2:**
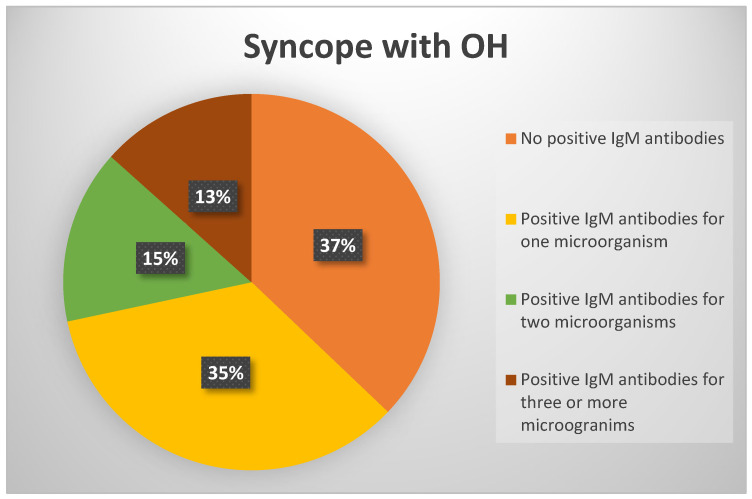
Positive IgM antibodies for group 2.

In group 2, 72 subjects (37.1%) tested negative for all IgM antibodies, while 122 (62.9%) had positive IgM antibodies for at least one microorganism (Figure 2).

The highest percentage was for IgM antibodies to Coxsackievirus (n = 42, 21.6%) (Table 3 and Table 4).

**Figure 3:**
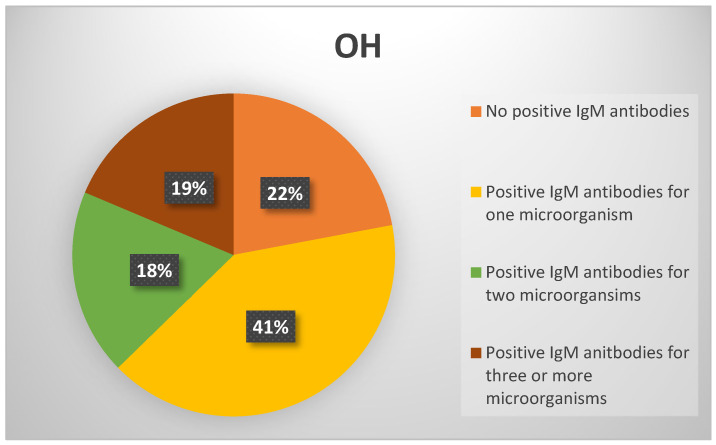
Positive IgM antibodies for group 3.

In group 3, 13 subjects (22%) tested negative for IgM antibodies, while 46 (78%) were positive for at least one microorganism (Figure 3).

The highest percentage was for IgM antibodies to Coxsackievirus (n = 17, 28.8%) (Table 3 and Table 4).

IgM antibodies to *Mycoplasma pneumoniae* were more prevalent in group 3 (n = 9, 15.3%) than in group 2 (n = 13, 6.7%; *p* < 0.05) (Table 3). IgM antibodies to CMV were more common in group 3 (n = 11, 18.6%) than in group 1 (n = 19, 7.9%). Group 2 also had a higher proportion of positive IgM antibodies for CMV (n = 21, 10.8%) than group 1, but the difference was not statistically significant (*p* > 0.05) (Table 4). Regarding IgM antibodies to EBV, all groups differed significantly (*p* < 0.05), with the highest percentage in group 3 (n = 8, 13.6%) and the lowest in group 1 (n = 4, 1.7%). IgM antibodies to HSV1 were more prevalent in group 1 (n = 30, 12.4%) than in group 2 (n = 11, 5.7%; *p* < 0.05) and group 3 (n = 4, 6.8%; *p* > 0.05).

### 3.3. Binary Logistic Regression

For a positive HUTT, multivariate analysis identified female gender (adjusted Exp(B) = 3.24; *p* = 0.000) and IgG antibodies to CMV (adjusted Exp(B) = 2.07; *p* = 0.01) as statistically significant predictors (Table 5).

For hypertensive reactions during the HUTT, male gender (adjusted Exp(B) = 2.35; *p* = 0.022) and positive IgM antibodies to Parvovirus B19 (adjusted Exp(B) = 4.78; *p* = 0.032) were significant predictors.

For OH during the HUTT, multivariate analysis identified female gender (adjusted Exp(B) = 1.92; *p* = 0.008), IgM antibodies to Coxsackievirus (adjusted Exp(B) = 2.11; *p* = 0.005), and IgM antibodies to EBV (adjusted Exp(B) = 4.36; *p* = 0.031) as statistically significant predictors (Table 6).

### 3.4. Multinomial Logistic Regression

Multinomial logistic regression was used to determine the likelihood of group membership based on hemodynamic changes (e.g., blood-pressure variations), age, sex, and serological results (IgM and IgG antibodies). Positive IgM antibodies to Parvovirus B19 were associated with a 5.1 times higher likelihood of belonging to group 1 (syncope) than group 2 (syncope with OH) (*p* < 0.05) (Table 7).

Positive IgM antibodies to Coxsackievirus were associated with a 3.45 times higher likelihood of belonging to group 2 (syncope with OH) than group 1 (syncope) (*p* < 0.05).

Positive IgG antibodies to *Borrelia* spp. were associated with a 5.78 times higher likelihood of belonging to group 3 (OH) than group 1 (syncope) (*p* < 0.05). Positive IgM antibodies to Coxsackievirus indicated a 6.85 times higher likelihood of belonging to group 3 (OH) than group 1 (syncope) (*p* < 0.05). Positive IgG antibodies to *Bartonella henselae* were associated with a 16.39 times higher likelihood of belonging to group 3 (OH) than group 2 (syncope with OH) (*p* < 0.05) (Table 7).

## 4. Discussion

The positivity of the HUTT in our study among patients with syncope without OH was higher than in the study by Esquivias et al. (61.16% vs. 25.4%) [21]. Noormand et al. emphasized the influence of age on hemodynamic changes during the head-up tilt test, linking the cardioinhibitory response to a younger population [22]. In our study, we focused on the hemodynamic patterns that precede the definitive mechanism of loss of consciousness. Forty-seven percent of patients with neurally mediated syncope experienced extreme pressure variations, likely caused by periodic sympathetic inhibition and vagus activation, a pattern termed “syncope with latency” [20]. Julu et al. described similar changes before presyncope during the HUTT, with an average amplitude of variation of 27 ± 3.1 mmHg and a mean duration of 10.6 ± 0.3 s [23]. Hausenloy et al. found that blood-pressure oscillations ≥ 30 mmHg had an 87% positive predictive value for the occurrence of vasovagal syncope during the HUTT [24]. In our study, 7.5% of patients exhibited an extreme hypertensive reaction during the HUTT, likely indicating decreased baroreflex sensitivity in the supine position, as also observed by Petersen et al. [25].

In patients with OH, 34–53%, depending on the group, exhibited extreme pressure variations before or after OH, indicating complex hemodynamic changes. Additionally, subjects with OH were more likely to experience small blood-pressure oscillations in the supine position compared with patients with syncope without OH. In our study, multivariate binary regression analysis showed that the presence of small variations in supine blood pressure indicated a 4.76 times lower chance of a positive HUTT result [26]. In patients with OH, in addition to the amplitude of the pressure drop, maintaining cerebrovascular autoregulation is crucial for preventing syncope [26].

The novelty of this study lay in the results of the serological tests. None of the subjects exhibited signs of an active infection at the time of testing. Some respondents reported their first episode of syncope several weeks to months after experiencing “flu-like” symptoms. In this context, positive IgM antibodies were employed as indicators of recent or ongoing infection, while IgG antibodies were utilized to assess immunological memory. This dual approach aimed to elucidate the potential role of subclinical infections in disrupting autonomic regulation, thereby contributing to the observed hemodynamic abnormalities.

Among patients with syncope but without OH, 57.85% had positive IgM antibodies to at least one microorganism, with 15% having positive IgM antibodies to two microorganisms and 10% to three or more. The highest percentage of positive IgM antibodies was for *Borrelia* spp. (14.5%). These patients did not exhibit signs of atrioventricular (AV) block on repeated ECG monitoring, excluding cardiogenic syncope and the need for pacemaker implantation.

In the group with syncope and OH, 63% had positive IgM antibodies, with 15% positive for two microorganisms and 10% for three or more. The group with only OH, without a history of syncope, had the highest percentage of positive IgM antibodies (78%), including the highest percentages of IgM antibodies for two microorganisms (18%) and for three or more microorganisms (19%).

Positive IgM antibodies to EBV and CMV were more common in both groups with OH than in patients with neurally mediated syncope, whereas IgM antibodies to HSV were more common in the syncope group. Interestingly, the percentage of IgG antibodies to HSV1, VZV, CMV, and EBV was highest in the syncope group compared with the other two groups (Table 4). However, for antibodies to Coxsackievirus, both IgM and IgG antibodies were more frequent in group 3 (OH). Regarding bacterial IgM antibodies, the highest percentage of positive antibodies to *Mycoplasma pneumoniae* was in group 3 (OH). Similar to viral IgG antibodies, IgG antibodies to *Mycoplasma pneumoniae* were more prevalent in group 1 (Table 3). Additionally, IgG antibodies to *Coxiella burnetii* were more frequent in group 1. Although not statistically significant, IgG antibodies to *Bartonella* were also highest in group 1 compared with the other two groups. In contrast, IgG antibodies to *Yersinia enterocolitica* were most common in group 3 (OH) (Table 3).

Multivariate analysis demonstrated that certain IgM antibodies significantly increased the likelihood of specific hemodynamic responses during the HUTT. For example, positive IgM antibodies to Parvovirus B19 were associated with a 4.78 times greater chance of a hypertensive reaction during the HUTT. Positive IgM antibodies to Coxsackievirus were linked to a 3.06 times higher chance of small blood-pressure oscillations during the HUTT. In contrast, positive IgG antibodies to Coxsackievirus indicated a 2.55 times greater likelihood of extreme pressure fluctuations during the HUTT.

In univariate analysis, many IgM and IgG antibodies showed significant associations with OH during the HUTT. After multivariate analysis, IgM antibodies to Coxsackievirus and EBV were associated with a 3.39 and 4.36 times greater likelihood of OH, respectively. Conversely, IgM antibodies to HSV were linked to a 2.17 times lower chance of OH. IgG antibodies to Adenovirus and VZV were associated with a 1.54 and 2.33 times lower chance of developing OH during the HUTT, respectively.

Multinomial regression analysis showed that positive IgG antibodies to *Borrelia* spp. significantly increased the likelihood of belonging to group 3 (OH only) compared with the other two groups with syncope. This finding aligns with previous studies reporting orthostatic intolerance in patients with Lyme disease [6]. Although IgG antibodies to *Bartonella* were more frequent in group 1, multinomial regression analysis showed that positive IgG antibodies increased the likelihood of belonging to group 3 (OH only) by 16.39 times, without a history of syncope during the HUTT.

Sporadic cases of autonomic dysfunction during or after acute infections with microorganisms have been reported in the literature [18,27,28,29,30,31,32,33,34,35,36,37]. Nakao et al. observed the occurrence of OH in active CMV infections, consistent with our findings of more frequent positive IgM antibodies to CMV in both OH groups compared with the syncope group without OH [34]. Gravelsina et al. identified a relationship between increased HHV6B viral load and autoantibodies to autonomic receptors in patients with chronic fatigue syndrome [38]. Additionally, studies on animal models have reported associations between infections and autonomic dysfunction, particularly for West Nile virus, HSV2, and Coxsackievirus [39,40,41,42]. Notably, hypotheses suggest that autonomic ganglia may serve as a site of latent infection with HSV and VZV, with possible reactivation [16,43].

Furthermore, with regard to Coxsackievirus and the ANS, Mathuranath et al. explored the role of this virus in the development of autonomic dysfunction following mumps [44]. In a case study by Pavesi et al., axonal degeneration with moderate loss of myelinated fibers (primarily small fibers) was observed in patients with Coxsackievirus infection and acute pandysautonomia, suggesting either immune-mediated mechanisms or direct viral action [37]. Additionally, in animal models infected with Coxsackievirus B1, lesions were found in peripheral autonomic ganglia [45]. In the case of EBV, Besnard et al. documented acquired hypoganglionosis due to EBV reactivation, while Bennett et al. reported acute autonomic neuropathy with confirmed EBV infection in cerebrospinal fluid (CSF) [33,46]. These findings align with the results of this study, indicating that both acute infections and reactivation of EBV can lead to ANS dysfunction.

It is important to note that viral infections, such as those caused by Coxsackievirus, CMV, and HHV6B, are not only implicated in autonomic dysfunction but also represent one of the leading causes of myocarditis [47]. Myocarditis itself is often associated with subtle clinical manifestations, including autonomic disturbances, and may serve as an intermediary link between infections and conditions such as syncope or orthostatic hypotension. Beyond viral etiologies, bacterial pathogens such as *Borrelia* spp., *Mycoplasma pneumoniae*, and *Chlamydia pneumoniae*, as well as parasitic infections such as *Trypanosoma cruzi* (Chagas Disease) have been recognized as triggers of myocarditis [48]. For instance, myocarditis associated with Lyme disease affects approximately 50% of patients with myocardial changes, and 15% may develop conduction disorders requiring pacemaker implantation [49].

These findings highlight the need to consider the potential role of myocarditis and subclinical infections in the diagnostic evaluation of patients presenting with syncope or OH. The subtle interplay between infections, autonomic dysfunction, and myocardial involvement underscores the complexity of the pathophysiological mechanisms underlying these conditions.

### Study Limitations

This observational cross-sectional retrospective review study lacked a control group of healthy subjects with serological tests, which would have allowed for comparisons with the study groups.

## 5. Conclusions

Our study found that the majority of patients with syncope and OH had positive IgM antibodies to a variety of microorganisms. Multivariate analysis also showed that positive IgM and IgG antibodies had predictive value for HUTT outcomes. Based on our findings, we hypothesize that infection may play a role in the pathophysiology of syncope and OH. Further studies are needed to elucidate the exact mechanisms involved and should also include a healthy control group and consider sex and age matching. Furthermore, longitudinal studies should also be considered to track parameters obtained through various functional diagnostics of the ANS, such as cardiovascular reflex tests, heart-rate variability, and HUTTs, alongside serological findings. These studies should also investigate the recurrence of syncope before and after specific medical interventions, such as pharmacological treatments, to better understand the potential therapeutic implications.

## Figures and Tables

**Table 1:** Demographic characteristics of study population.

	(1) Syncope N = 506	(2) Syncope with OH N = 235	(3) OH N = 65	*p* Value
Male (n, %)	146 (28.9%) ^2^	50 (21.3%) ^1,3^	22 (33.8%) ^2^	<0.05 ^a^
Female (n, %)	360 (71.1%) ^2^	185 (78.7%) ^1,3^	43 (66.2%) ^2^	
Age (mean ± SD)	45.68 ± 16.03	47.67 ± 15.16	45 ± 13.35	>0.05 ^b^
Hypertension (n, %)	105 (20.8%)	32 (13.6%)	8 (12.3%)	0.003 ^a^
DM (n, %)	27 (5.3%)	7 (3%)	0	0.002 ^a^
ME/CFS (n, %)	107 (21.1%)	72 (30.6%)	29 (44.6%)	<0.001 ^a^

^a^—chi-squared test; ^b^—one-way ANOVA; DM—diabetes mellitus; ME/CFS—myalgic encephalomyelitis/chronic fatigue syndrome; numerical superscript—significant difference between the examined group and group named by superscript.

**Table 2:** Results from HUTT.

Groups	Syncope (n = 506)	Syncope with OH (n = 235)	OH (n = 65)	Sig.
n (%)				
Positive head-up tilt test	30 (61.6%)	213 (90.6%)	0(0.0%)	*p* ^1^ < 0.001 *p* ^2^ < 0.001 *p* ^3^ < 0.001
Extreme variation in blood pressure—supine position, passive phase	238 (47%)	79 (33.6%)	35 (53.8%)	*p* ^1^ < 0.001 *p* ^2^ > 0.05 *p* ^3^ < 0.001
Small variation in blood pressure—supine position	24 (4.7%)	26 (11.1%)	8 (12.3%)	*p* ^1^ < 0.01 *p* ^2^ < 0.05 *p* ^3^ >0.05
Small variation in blood pressure—supine position, passive phase	50 (9.9%)	28 (11.9%)	19 (29.2%)	*p* ^1^ > 0.001 *p* ^2^< 0.05 *p* ^3^ < 0.001
Hypertensive reaction	91 (18.00%)	3 (1.3%)	3 (4.6%)	*p* ^1^ < 0.001 *p* ^2^ > 0.01 *p* ^3^ > 0.05
Extreme hypertensive reaction	38 (7.5%)	0 (0.0%)	1 (1.5%)	*p* ^1^ < 0.001 *p* ^2^ > 0.05 *p* ^3^ > 0.05
POTS	40 (7.9%)	0 (0%)	0 (0.0%)	*p* ^1^ < 0.001 *p* ^2^ < 0.001 *p* ^3^ > 0.05
Hypotension in supine position	12 (2.4%)	20 (8.5%)	2 (3.1%)	*p* ^1^ < 0.001 *p* ^2^ > 0.05 *p* ^3^ > 0.05

*p* ^1^—*p* value for comparison between group 1 (syncope) and group 2 (syncope with OH); *p* ^2^—*p* value for comparison between group 1 (syncope) and group 3 (OH); *p* ^3^—*p* value for comparison between group 2 (syncope with OH) and group 3 (OH).

**Table 3:** Results of positive IgM and IgG antibodies for bacteria among the groups.

Group	Syncope (n = 242)	Syncope with OH (n = 194)	OH (n = 59)	Sig.
Positive IgM and IgG Antibodies (n, %)				
IgM Mycoplasma pneumoniae	18 (7.4%)	13 (6.7%)	9 (15.3%)	*p* ^1^ > 0.05 *p* ^2^ > 0.05 *p* ^3^ < 0.05
IgG Mycoplasma pneumoniae	97 (40.1%)	37 (19.1%)	15 (25.4%)	*p* ^1^ < 0.001 *p* ^2^ < 0.05 *p* ^3^ > 0.05
IgG Coxiella burnetii	15 (6.2%)	4 (2.1%)	0 (0%)	*p* ^1^ < 0.05 *p* ^2^ < 0.05 *p* ^3^ > 0.05
IgG Yersinia enterocolitica	7 (2.9%)	5 (2.6%)	6 (10.2%)	*p* ^1^ > 0.05 *p* ^2^ < 0.05 *p* ^3^ < 0.05
IgG Bartonella henselae	16 (6.6%)	3 (1.5%)	3 (5.1%)	*p* ^1^ < 0.05 *p* ^2^ > 0.05 *p* ^3^ > 0.05

*p* ^1^—*p* value for comparison between group 1 (syncope) and group 2 (syncope with OH); *p* ^2^—*p* value for comparison between group 1 (syncope) and group 3 (OH); *p* ^3^—*p* value for comparison between group 2 (syncope with OH) and group 3 (OH).

**Table 4:** Results of positive IgM and IgG antibodies for viruses among the groups.

Group	Syncope (n = 242)	Syncope with OH (n = 194)	OH (n = 59)	Sig.
Positive IgM and IgG Antibodies (n, %)				
IgG Adenovirus	147 (60.7%)	65 (33.5%)	23 (39%)	*p* ^1^ < 0.001 *p* ^2^ < 0.01 *p* ^3^ > 0.05
IgM Coxsackievirus	8 (3.3%)	42 (21.6%)	20 (33.9%)	*p* ^1^ < 0.001 *p* ^2^ < 0.001 *p* ^3^ > 0.05
IgG Coxsackievirus	16 (6.6%)	34 (17.5%)	17 (28.8%)	*p* ^1^ < 0.001 *p* ^2^ < 0.001 *p* ^3^ > 0.05
IgM CMV	19 (7.9%)	21 (10.8%)	11 (18.6%)	*p* ^1^ < 0.05 *p* ^2^ < 0.001 *p* ^3^ < 0.05
IgG CMV	187 (77.3%)	135 (69.6%)	32 (54.2%)	*p* ^1^ > 0.05 *p* ^2^ < 0.001 *p* ^3^ < 0.05
IgM EBV	4 (1.7%)	11 (5.7%)	8 (13.6%)	*p* ^1^ < 0.05 *p* ^2^ < 0.001 *p* ^3^ < 0.05
IgG EBV	190 (78.5%)	139 (71.6%)	32 (54.2%)	*p* ^1^ > 0.05 *p* ^2^ < 0.001 *p* ^3^ < 0.05
IgG VZV	191 (78.9%)	88 (45.4%)	26 (44.1%)	*p* ^1^ < 0.001 *p* ^2^ < 0.001 *p* ^3^ > 0.05
IgM HSV1	30 (12.4%)	11 (5.7%)	4 (6.8%)	*p* ^1^ < 0.05 *p* ^2^ > 0.05 *p* ^3^ > 0.05
IgG HSV1	147 (60.7%)	61 (31.4%)	17 (28.8%)	*p* ^1^ < 0.001 *p* ^2^ < 0.001 *p* ^3^ > 0.05

*p* ^1^—*p* value for comparison between group 1 (syncope) and group 2 (syncope with OH). *p* ^2^—*p* value for comparison between group 1 (syncope) and group 3 (OH). *p* ^3^—*p* value for comparison between group 2 (syncope with OH) and group 3. (OH).

**Table 5:** Binomial logistic regression for outcomes during HUTT.

Positive Head-Up Tilt Test				
	Univariate		Multivariate	
Parameter	Sig.	Exp (B)	Sig.	Adjusted Exp (B)
Sex (Female)	0.000 ***	3.44 (2.05–5.44)	0.000 ***	3.24 (1.95–5.39)
Small variation in blood pressure at supine position	0.000 ***	0.17 (0.07–046)	0.002 **	0.21 (0.08–0.56)
IgG-positive CMV	0.004 **	2.14 (1.28–3.56)	0.01 *	2.07 (1.19–3.6)
IgG-positive Mycoplasma pneumoniae	0.038 *	0.62 (0.4–0.97)	0.049 *	0.61(0.38–0.98
IgG-positive SARS-CoV 19	0.02 *	0.45 (0.23–0.88	0.094	0.54 (0.26–1.11)
Extreme Variation in Blood Pressure During HUTT				
Sex (male)	0.009 **	1.89 (1.18–3.03)	0.011 *	1.89 (1.16–3.03)
IgG-Positive Coxsackievirus	0.02 *	2.65 (1.17–6.02)	0.029 *	2.55 (1.1–5.89)
IgG-Positive CMV	0.011 *	0.52 (0.31–0.86)	0.039 *	0.58 (0.34–0.97)
IgG-Positive Chlamydia pneumoniae	0.041	0.63 (0.4–0.98)	0.112	0.69 (0.43–1.09)
Small Variation in Blood Pressure During HUTT				
IgM-Positive Coxsackievirus	0.04 *	3.06 (1.06–12.28)	0.04 *	3.06 (1.06–12.28)
Hypertensive Response				
Sex (Male)	0.028 *	2.25 (1.09–4.63)	0.022 *	2.35 (1.13–4.88)
Positive IgM Parvo B19 virus	0.042 *	4.27 (1.05–17.36)	0.032 *	4.78 (1.15–19.93)
POTS				
Age	0.001 **	0.94 (0.91–0.98)	0.001 **	0.94 (0.91–0.98)

* *p* < 0.05; ** *p* < 0.01; *** *p* < 0.001.

**Table 6:** Binomial logistic regression analysis for appearance of OH during HUTT.

OH				
	Univariate		Multivariate	
Parameter	Sig.	Exp (B)	Sig.	Adjusted Exp (B)
Sex (Female)	0.021 *	1.61 (1.07–2.42)	0.008 **	1.92 (1.19–3.13)
IgG-positive Adenovirus	0.000 ***	0.34 (0.24–0.5)	0.08 *	0.65 (0.41–1.05)
IgM-positive Coxsackievirus	0.000 ***	9.46 (4.42–20.25)	0.005 **	3.39 (1.44–7.96)
IgG-positive Coxsackievirus	0.000 ***	3.99 (2.14–7.43)	0.051	2.11 (1–4.47)
IgG-positive CMV	0.003 **	0.55 (0.38–0.82)	0.711	0.91 (0.54–1.53)
IgM-positive EBV	0.004 **	6.05 (1.76–20.83)	0.031 *	4.36 (1.15–16.6)
IgG-positive EBV	0.008 **	0.58 (0.38–0.86)	0.691	1.12 (0.65–1.94)
IgG-positive Mycoplasma pneumoniae	0.000 ***	0.4 (0.27–0.6)	0.166	0.72 (0.45–1.15)
IgM-positive Coxiella burnetti	0.017 *	0.44 (0.23–0.86)	0.12	0.52 (0.23–1.19)
IgG-positive Coxiella burnetti	0.012 *	0.24 (0.08–0.74)	0.295	0.5 (0.14–1.82)
IgA-positive Helicobacter pylori	0.012 *	0.43 (0.22–0.83)	0.061	0.48 (0.22–1.03)
IgG-positive VZV	0.000 ***	0.23 (0.15–0.34)	0.001 **	0.43 (0.26–0.7)
IgM-positive HSV1	0.008 **	0.41 (0.21–0.79)	0.043 *	0.46 (0.22–0.98)
IgG-positive HSV1	0.000 ***	0.3 (0.2–0.43)	0.084	0.66 (0.41–1.06)
IgM-positive Bartonella henselae	0.000 ***	0.3 (0.08–0.8)	0.303	0.51 (0.14–1.85)
IgG-positive Bartonella henselae	0.027 *	0.4 (0.13–0.88)	0.491	0.68 (0.23–2.04)

* *p* < 0.05; ** *p* < 0.01; *** *p* < 0.001.

**Table 7:** Multinomial regression analysis between the groups.

Reference Category: Group 1 (Syncope)					
		Sig.	Exp(B)	95% Confidence Interval for Exp(B)	
				Lower Bound	Upper Bound
Group 2 (syncope with OH)	[AdenoIgG = negative]	0.046 *	1.731	1.009	2.970
	[AdenoIgG = positive]	0 *.			
	[ParvoIgM = negative]	0.020 *	5.096	1.292	20.102
	[ParvoIgM = positive]	0 *.			
	[KoksIgM = negative]	0.010 *	0.290	0.114	0.741
	[KoksIgM = positive]	0 *.			
	[MikoPnIgG = negative]	0.026 *	1.830	1.074	3.116
	[MikoPnIgG = 1.00]	0 *.			
	[HerpesZosterIgG = negative]	0.000 ***	2.710	1.554	4.726
	[HerpesZosterIgG = positive]	0 *.			
	[Sex = male]	0.001 *	0.394	0.225	0.689
	[Sex = female]	0 *.			
Group 3 (OH)	[BorIgG = negative]	0.006 **	0.173	0.050	0.599
	[BorIgG1 = positive]	0 *.			
	[KoksIgM = negative]	0.001 *	0.146	0.045	0.471
	[KoksIgM = positive]	0 *.			
	[EBVIgM = negative]	0.041 *	0.206	0.045	0.938
	[EBVIgM = positive]	0 *.			
**Reference Category: Group 2 (Syncope with OH)**					
Group 3 (OH)	[BorIgG = negative	0.029 *	0.251	0.073	0.869
	[BorIgG = positive	0 *.			
	[BartonelaIgG = negative	0.008 **	0.061	0.008	0.482
	[Bartonela_IgG = positive	0 *.			

0 *—the parameter is set to zero because its redundant. * *p* < 0.05; ** *p* < 0.01; *** *p* < 0.001.

## Data Availability

Data are available upon reasonable request.
